# Laryngospasm Complicating Awake Nasal Intubation in Ludwig's Angina

**DOI:** 10.1002/ccr3.71772

**Published:** 2025-12-30

**Authors:** Sara Onuki, Yoshihiro Hayakawa, Naoki Yonezawa

**Affiliations:** ^1^ Department of Emergency and Critical Care Medicine Yokohama City Minato Red Cross Hospital Yokohama Kanagawa Japan

**Keywords:** airway management, deep neck infection, laryngospasm, Ludwig's angina

## Abstract

A 64‐year‐old man with Ludwig's angina developed laryngospasm during awake fiberoptic intubation despite mild supraglottic edema. Paralysis restored ventilation and allowed successful intubation. This case illustrates that even minimal laryngeal inflammation can trigger reflex airway closure, underscoring the need for preparedness for failed awake intubation and potential surgical airway intervention.

## Graphical abstract

Axial and sagittal contrast‐enhaced computed tomography images demonstrating Ludwig's angina with mild supraglottic edema. Even minimal laryngeal inflammation can precipitate laryngospasm during awake airway management.

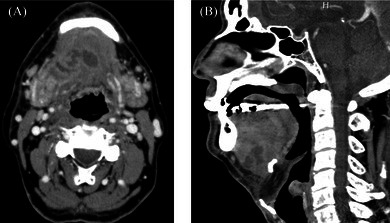

## 1 Case Report

**FIGURE 1:**
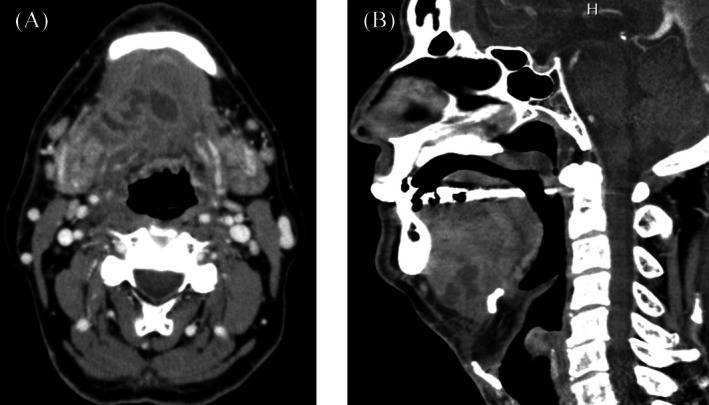
(A) Axial contrast‐enhanced computed tomography showing fluid collections in the floor of the mouth and submandibular spaces. (B) Sagittal contrast‐enhanced computed tomography demonstrating posterior displacement of the tongue due to soft tissue swelling in the submandibular and sublingual spaces.

**FIGURE 2:**
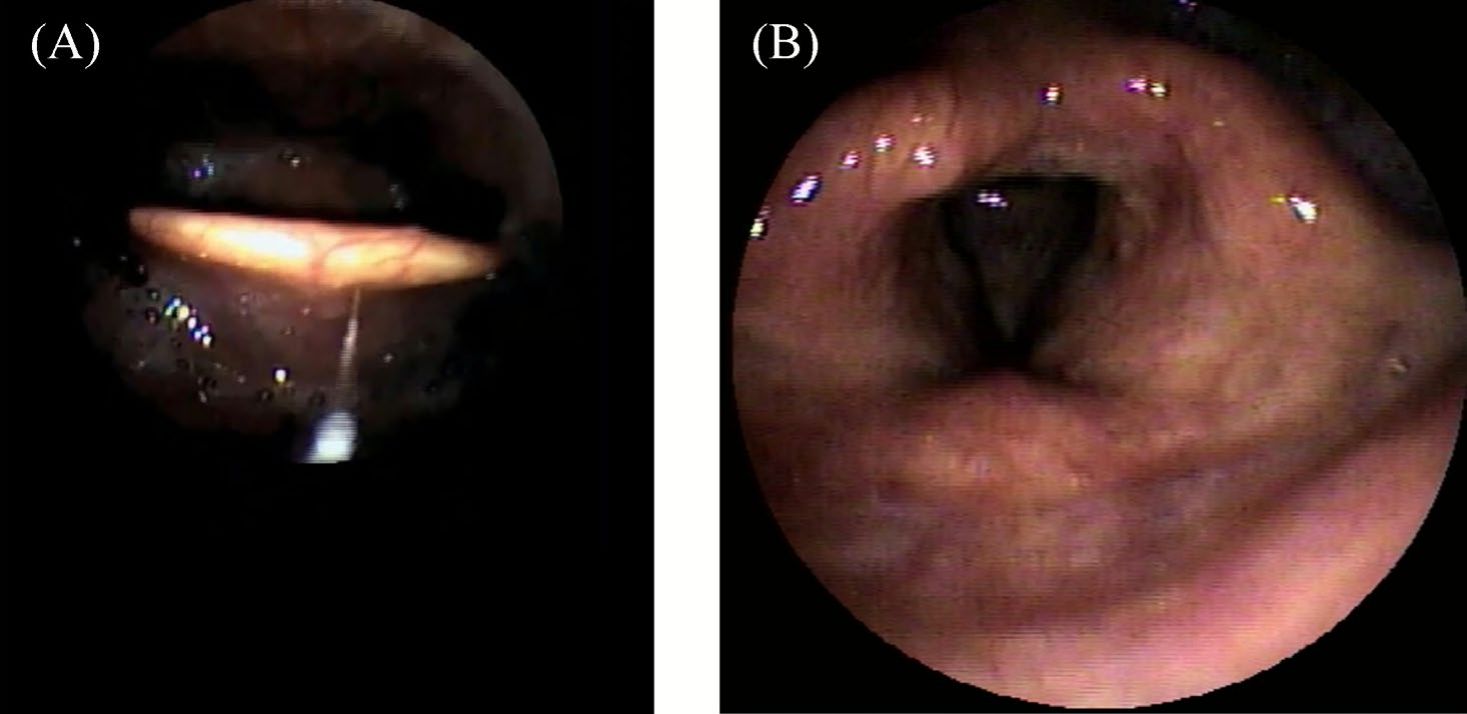
Fiberoptic laryngoscopy revealing mild edema of the epiglottis (A) and supraglottic structures (B).

A 64‐year‐old man (165 cm, 59 kg) presented with neck swelling and trismus 5 days after treatment for mandibular molar caries. Examination revealed submandibular‐to‐anterior cervical induration with erythema, sublingual edema, and an interincisal distance < 2 fingerbreadths; notably, stridor was absent. Contrast‐enhanced computed tomography of the neck confirmed Ludwig's angina, showing fluid collections in the mouth floor and submandibular spaces with posterior tongue displacement (Figure 1A,B). Fiberoptic laryngoscopy revealed mild edema of the epiglottis and supraglottic structures (Figure 2A,B). Given the risk of rapid airway compromise before definitive surgery, awake nasotracheal intubation under bronchoscopic guidance was attempted with cricothyrotomy backup. After administering intravenous fentanyl (150 μg), the glottis was briefly visualized before severe coughing, secretions, and laryngeal contraction obscured the view upon contact with edematous supraglottic tissue. Propofol was administered in two divided doses to a total of 100 mg, but visualization did not improve over 5 min. The patient then became unventilatable, prompting the withdrawal of the bronchoscope and the initiation of manual ventilation. Rocuronium (20 mg) restored ventilatability immediately; an additional 20 mg enabled successful bronchoscope‐guided nasotracheal intubation with a 6.5‐mm spiral tube following revisualization of the glottis. Oxygenation (SpO_2_ > 90%) was maintained throughout the ~20 min airway management period. Based on the overall clinical course, laryngospasm was suspected on clinical assessment. On day 3, surgical drainage of the abscess and tracheostomy were performed. The patient recovered uneventfully and was discharged home on day 17.

## 2 Discussion

Ludwig's angina is a rapidly progressive infection of the floor of the mouth and submandibular spaces that can lead to airway obstruction. Inflammation involving the masticatory muscles can distort airway anatomy, limit mouth opening, and exacerbate intraoral swelling, complicating airway management. Although awake nasotracheal intubation is generally preferred in these patients, this case highlights an additional physiological risk—laryngospasm triggered by laryngeal inflammation. Laryngospasm is a potentially life‐threatening condition characterized by involuntary contraction of the laryngeal muscles, resulting in sustained partial or complete glottic obstruction and typically manifests with acute dyspnea and stridor, potentially progressing to hypoxemia, bradycardia, or death. Although commonly recognized as an anesthesia‐related adverse event—particularly during or after extubation in pediatric patients [1]—several reports describe its occurrence in Ludwig's angina as well [2]. Beyond anatomic challenges such as trismus and tongue displacement, supraglottic edema, secretions, and severe coughing provoked by mucosal contact may induce vagally mediated laryngospasm and abrupt airway loss. Because direct visualization is often difficult and diagnosis relies heavily on clinical assessment, its true incidence in severe inflammatory conditions, including Ludwig's angina, may be underestimated [3]. Although neuromuscular blockade facilitated airway control in this case, its use in Ludwig's angina should be reserved for carefully controlled conditions because of the risk of catastrophic airway collapse due to anatomical distortion. Notably, laryngospasm may occur even when visible inflammation appears mild, emphasizing the need for surgical backup.

## Author Contributions

**Sara Onuki:** conceptualization, data curation, writing – original draft. **Yoshihiro Hayakawa:** conceptualization, data curation, writing – original draft. **Naoki Yonezawa:** supervision, writing – review and editing.

## Funding

The authors have nothing to report.

## Consent

Written informed consent was obtained from the patient for this publication of this case report and accompanying images, in accordance with journal guidelines.

## Conflicts of Interest

The authors declare no conflicts of interest.

## Data Availability

The authors have nothing to report.
